# Source Apportionment and Health Risk Assessment of Heavy Metals in Endemic Tree Species in Southern China: A Case Study of *Cinnamomum camphora* (L.) Presl

**DOI:** 10.3389/fpls.2022.911447

**Published:** 2022-07-11

**Authors:** Ning Li, Yan Li, Shenglu Zhou, Huanchao Zhang, Genmei Wang

**Affiliations:** ^1^College of Forestry, Nanjing Forestry University, Nanjing, China; ^2^Key Laboratory of Geographic Information Science of the Ministry of Education, School of Geographic Sciences, East China Normal University, Shanghai, China; ^3^School of Geography and Ocean Science, Nanjing University, Nanjing, China

**Keywords:** camphor tree, heavy metals, health risk, Pb isotope, source apportionment

## Abstract

As a developed economic region in China, the problem of heavy metals (HMs) pollution in the Yangtze River Delta has become increasingly prominent. As an important evergreen broad-leaved tree species in southern China, the camphor tree cannot only be used as a street tree but also its various tissues and organs can be used as raw materials for Chinese herbal medicine. In order to explore whether heavy metal contamination in the region threatens the safety of camphor trees as pharmaceutical raw materials, we collected the bark and leaves of the tree most commonly used for pharmaceuticals in Yixing City. Based on the determination of HMs content, the health risks after human intake are evaluated, the sources and contributions of HMs are analyzed, and then the health risks of pollution sources are spatially visualized. The results showed that under the influence of human activities, the camphor trees in the study area had obvious enrichment of HMs, and the over-standard rate of Pb in the bark was as high as 90%. The non-carcinogenic risks of bark and leaves are acceptable, but the carcinogenic risks are not acceptable. The bark had the highest average carcinogenic risk, approaching six times the threshold. The results of Pb isotope ratio analysis showed that the average contribution rate of industrial activities to HMs in camphor trees in the study area was the highest, reaching 49.70%, followed by fossil fuel burning (37.14%) and the contribution of natural sources was the smallest, only 13.16%. The locations of the high-risk areas caused by the three pollution sources in the study area are basically similar, mainly concentrated in the northwest, northeast, and southeast, which are consistent with the distribution of industries and resources in the study area. This study can provide a reference for the precise prevention of HMs pollution of camphor and the safe selection of its pharmaceutical materials.

## Introduction

In recent years, with the rapid development of industrialization and urbanization, the accumulation of heavy metals (HMs) in the environment has been increasing, especially in developed economic regions (Yang S. H. et al., 2020). HMs are highly toxic, refractory, and persistent pollutants. They will enter the soil through various means such as atmospheric deposition, surface runoff, and sewage irrigation, then get absorbed by plants, and finally enter the human body through the food chain, thus threatening human health (Cui et al., 2022). At present, a large number of studies have paid attention to this problem, but most of these research objects are polluted soil, sediment, and atmospheric deposition (Men et al., 2020; Chen et al., 2021; Li et al., 2022). In addition, some researchers have conducted source analyses and health risk assessments of HMs in typical crops (Xiang et al., 2021; Zulkafflee et al., 2022). However, studies on HMs pollution of medicinal plants, especially medicinal higher woody plants, are relatively few.

The camphor tree is a subtropical evergreen species that has been cultivated in southern China for more than 1,500 years (Chen et al., 2020). The canopy of this tree stretches and its branches and leaves are luxuriant, making it an excellent tree for the streets and soundproof forest belts. Camphor trees also have important medicinal values. Its bark, roots, stems, leaves, and fruits are all rich in terpenoids, which have important pharmaceutical and industrial applications (Guo et al., 2016; Jiang et al., 2016). For example, D-borneol, a key ingredient in many traditional Chinese herbal formulas, is documented in multiple editions of the Chinese Pharmacopeia (Huang et al., 2016; Chai et al., 2019; Chen et al., 2019), can be used to treat cardiovascular diseases, including stroke, coronary heart disease, and angina pectoris (Yang Z. et al., 2020), and the market demand is very large. However, as HMs pollution in cities becomes more and more serious, are camphor trees also affected? A study compared the HMs accumulation capacity of six plants frequently seen on both the sides of the road and found that the camphor tree had the highest HMs accumulation index (Zhai et al., 2016), which intensified people’s concerns about the safety of camphor tree bark or leaves as medicinal raw materials. Therefore, it is necessary to carry out a health risk assessment and pollution source apportionment of HMs in camphor trees.

Yixing City is located in the southwest of Jiangsu Province, west of Taihu Lake, and in the center of the Shanghai–Nanjing–Hangzhou triangle. There are many factories, convenient transportation, and a dense population in this area. Therefore, economic development is good, but the problem of environmental pollution is becoming more and more prominent. Dingshu Town on the west bank of Taihu Lake is the concentrated distribution area of pottery factories in Yixing City. These factories produce a lot of HMs pollution during the production process (Li et al., 2018). The cadmium content of “Cadmium Red” and “Cadmium Yellow,” the raw materials for producing ceramics, is as high as 40 g/kg (Lin et al., 2015). Dingshu Town and Hufu Town are the concentrated distribution areas of camphor trees. HMs pollution in these areas may pose a threat to camphor trees and human health. Therefore, Dingshu Town and Hufu Town in Yixing City were selected as the study areas of this study.

The objectives of this study are: (1) to evaluate the health risks of HMs exposure in camphor tree bark and leaves to humans, (2) to analyze the source and contribution rate of HMs pollution, (3) to quantify source health risks by combining source apportionment with health risk assessment, and (4) to conduct a spatial analysis of source health risks. Our research results will help to effectively identify the high-risk areas caused by different HMs pollution sources to camphor trees and provide a reference for the priority treatment of different HMs pollution sources.

## Materials and Methods

### Sample Collection and Determination

**FIGURE 1:**
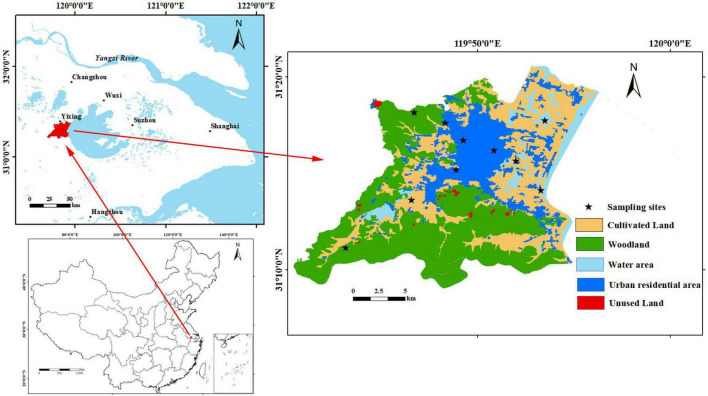
Study area and sampling sites.

The study area is located in Dingshu Town and Hufu Town, Yixing City, in the Yangtze River Delta, China. We set up 10 sampling sites to cover all the land use types in the study area (Figure 1). A mixture of bark and leaf samples from three camphor trees and the corresponding six soil samples from the surrounding areas were collected at each sampling site. The samples were brought back to the laboratory in time for drying and grinding, and 100 mg was weighed for digestion. Guaranteed reagents such as HNO_3_ and HClO_4_ were used to digest HMs by graphene electric hot plate heating method. HMs content was determined using an inductively coupled plasma mass spectrometry (ICP-MS) (PerkinElmer SCIEX, Elan 9000). Blank samples and parallel samples were used for quality control.

The Pb concentration of the sample was diluted to approximately 30 ng/ml and the ratios of ^208^Pb/^206^Pb and ^206^Pb/^207^Pb were determined using ICP-MS. The equipment was calibrated using standard reference material SRM981 [National Institute of Standards and Technology (NIST), Gaithersburg, MD, United States]. To ensure the precision and accuracy of the measurements, the NIST SRM981 was used for every two samples.

### Human Health Risk Assessment

When humans ingest Chinese herbal medicines made from camphor bark or leaves, the HMs in them may pose health risks to humans. This study uses the human health risk assessment model recommended by the United States Environmental Protection Agency (USEPA) to characterize the non-carcinogenic and carcinogenic risks (USEPA, 2011). The model calculation formula is as follows:


$$\text{ADI}=\frac{C_{i}\times I\,R\times E\,F\times E\,D}{B\,W\times A\,T}$$

where, ADI is the average daily exposure dose (mg/kg/day) of directly ingested HMs; C_*i*_ is the concentration of HMs i in Chinese herbal medicines with bark or leaves as raw materials (mg/kg); IR is the maximum daily intake of the Chinese herbal medicines rate (kg/d), is 0.015 kg/d (Shandong Province Standard (SPS), 2012); EF is exposure frequency (d/a); ED is exposure duration (a); BW is average body weight (kg); and AT is the average exposure time (d); the values of the above parameters are shown in Supplementary Table 1.


$$T\,H\,I=\sum H\,I_{i}=\sum \frac{A\,D\,I_{i}}{R\,f\,D_{i}}$$

$$T\,C\,R=\sum C\,R_{i}=\sum \left(A\,D\,I_{i}\times S\,F_{i}\right)$$

where, HI_*i*_ is the non-carcinogenic hazard index of heavy metal i, THI is the non-carcinogenic hazard index of all the HMs; RfD_*i*_ is the non-carcinogenic daily reference dose of heavy metal i; HI/THI < 1 indicates that the non-carcinogenic risk of HMs is acceptable, otherwise there is a non-carcinogenic risk, CR_*i*_ is the carcinogenic risk for heavy metal i, TCR is the total carcinogenic risk (TCR) for all the HMs, and SF is the carcinogenic slope factor. The RfD and SF values of different heavy metal elements are shown in Supplementary Table 2. The acceptable risk level of CR/TCR recommended by the USEPA is 10^–6^ to 10^–4^ and higher than 10^–4^ indicates a significant carcinogenic risk (USEPA, 2001).

### Source Apportionment Based on the Pb Isotopes Ratio Analysis

Isosource is a stable isotope analysis software that is often used to calculate source contributions. Compared with binary or ternary mixed models, the advantage is that even when there is only one isotopic system and more than three types of pollution sources, the model can still obtain the most likely range of ratios contributed by different pollution sources (Chen et al., 2018b).

Input the Pb isotope ratio of potential pollution sources in the study area and the Pb isotope ratio of each sample into Isosource and the calculation formula is as follows:


$$R_{m}=\sum_{i=1}^{n}P_{i}\,R_{i}$$

$$I=\sum_{i}^{n}R_{i}$$

where, R_*m*_ is the isotopic ratio, P_*i*_ is the percentage contribution of source i, and R_*i*_ is the isotopic ratio of source i. There are two isotopic ratios and six possible sources in this study (Supplementary Table 3).

## Results

### Characteristics of Heavy Metal Contents in Bark and Leaves of the Camphor Tree

The contents of different heavy metal elements in the bark and leaves of the camphor tree are shown in Table 1. The concentration of HMs in the bark is higher than that in leaves, which confirmed the findings of a study by Sawidis et al. (2011). The coefficient of variation (CV) of HMs in bark, except Cd, is higher than those of leaves; the CV of all the heavy metal elements in the bark is greater than 36%, which belongs to high variation; the CV of As, Cr, Ni, and Zn in leaves is between 16 and 36%, it belongs to moderate variation, while Cu, Cd, and Pb belong to high variation. A larger CV indicates a greater influence from human activities (coal burning, traffic burning, sewage irrigation, industrial activities, etc.) (Shi and Lu, 2018). According to the “Chinese Medicine—Limits of HMs in Chinese Herbal Medicine” published on the website of the International Organization for Standardization (ISO), the maximum reference values of Pb, As, and Cd in Chinese herbal medicine are 10.00, 4.00, and 2.00 mg/kg (International organization for standardization (ISO), 2015). It was determined that in all the samples, only the concentration of Pb in bark exceeded the limit value and the over-standard rate (OSR) was as high as 90.00%. Studies have shown that *Populus canescens* has a strong ability to accumulate Pb, which is an important reason for the high content of Pb in plants. However, high concentrations of Pb lead to a decrease in plant photosynthetic rate and biomass (Shi et al., 2021). As a potential carcinogen, Pb has been implicated in the etiology of many diseases, especially those related to the cardiovascular, renal, nervous system, and skeletal diseases (Järup, 2003). Some studies found that the average accumulation capacity of Black Locust, Poplar, and Ginkgo for HMs was Cd > Zn > Cu > Pb (Zhan et al., 2014) and the accumulation capacity of tea tree for Pb was also less than other HMs (She et al., 2020). Although the enrichment capacity of Pb is relatively small, in this study area, only the Pb in the bark has the largest variation coefficient (69.37%) and the highest OSR, which indicates that the camphor trees in the study area have been obviously polluted by Pb.

**TABLE 1:** Heavy metal content in bark and leaves of the camphor tree.

		As	Cr	Ni	Cu	Zn	Cd	Pb
Barks	Min (mg/kg)	0.68	1.38	1.38	5.66	28.60	0.18	2.62
	Max (mg/kg)	1.92	11.02	11.16	26.33	250.79	1.07	60.95
	Mean (mg/kg)	1.19	5.84	4.58	15.51	125.30	0.75	28.95
	SD	0.45	2.86	2.88	6.05	68.62	0.35	20.08
	CV (%)	37.80	49.00	62.97	39.00	54.76	47.44	69.37
	OSR (%)	0	/	/	/	/	0	90.00
Leaves	Min (mg/kg)	0.44	0.83	1.47	3.87	24.2	0.04	1.12
	Max (mg/kg)	1.02	2.12	3.18	12.82	48.39	0.24	4.7
	Mean (mg/kg)	0.78	1.59	2.09	6.72	33.34	0.10	2.33
	SD	0.19	0.37	0.52	2.56	8.13	0.06	1.03
	CV (%)	24.22	23.34	25.07	38.14	24.39	55.40	44.10
	OSR (%)	0	/	/	/	/	0	0
	ISO (mg/kg)	4.00	/	/	/	/	2.00	10.00

### Human Health Risk Assessment of Heavy Metals in the Camphor Tree

**FIGURE 2:**
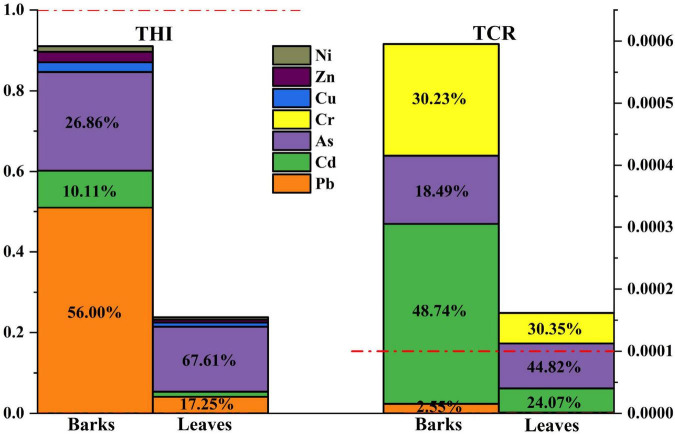
Contribution of heavy metals (HMs) to human total non-carcinogenic risk (THI) and total carcinogenic risk (TCR).

The total non-carcinogenic risk (THI) of HMs in the bark and leaves of the camphor tree in the study area was acceptable (THI < 1.0), but the TCR was unacceptable (TCR > 1 × 10^–4^) (Figure 2). For THI, Pb in bark contributed the most, reaching 56.00%, followed by As (26.86%); on the contrary, As in leaves contributed the most to THI, reaching 67.61%, followed by Pb (17.25%). Nonetheless, the THI of bark and leaves was still within the acceptable range, and it is worth noting that the THI of bark was already close to the threshold. For TCR, As in leaves still dominates, and the order of the contribution rate of HMs is As (44.82%) > Cr (30.35%) > Cd (24.07%) > Pb (0.76%); the contribution rate of HMs in bark was Cd (48.74%) > Cr (30.23%) > As (18.49%) > Pb (2.55%). Pb and Cd in the bark were the most likely to cause harm to humans, and similarly, As in leaves. Overall, the bark is more likely to be enriched with HMs than leaves, which poses a higher risk to human health and should be a major concern.

### Source Apportionment of Heavy Metals

The results of Pearson correlation analysis showed that Pb in bark showed a very strong correlation with Cu, Zn, and Cd, respectively (*r* > 0.8), Pb-Cr was strongly correlated, Pb-As was moderately correlated, and only Pb-Ni was very weak or had no correlation (Supplementary Figure 1). The stronger the correlation, the more likely these HMs are from the same pathway (Wang et al., 2020). Therefore, the source of Pb in the bark can reflect the source of most HMs. The correlation between Pb in leaves and other HMs is not as strong as that in the bark; however, Pb-Cr also shows a strong correlation (0.6 ≤ *r* < 0.8), Pb-As is moderately correlated (0.4 ≤ *r* < 0.6), and Pb-Ni and Pb-Zn are weakly correlated (0.2 ≤ *r* < 0.4). According to the analysis results in Figure 2, the HMs in the bark that has a larger impact on health risks are Pb, Cd, Cr, and As; similarly, As, Cr, Cd, and Pb in leaves have a larger impact on health risks.

**FIGURE 3:**
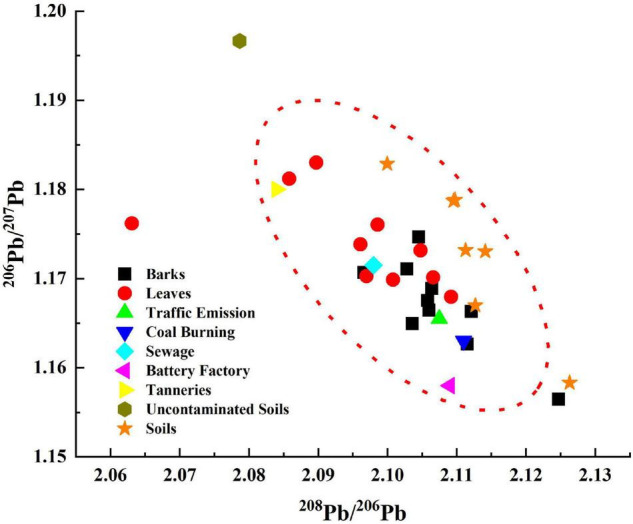
Comparison of Pb isotope composition between samples and potential pollution sources.

A comparison of Pb isotopic composition in barks and leaves with potential sources is shown in Figure 3. A total of 18 of the 20 samples as well as the corresponding soils had Pb isotope ratios close to five anthropogenic sources (traffic emissions, coal burning, sewage, battery factories, and tanneries). A total of 5 of the 6 corresponding soil samples were close to the anthropogenic sources mentioned above. However, the Pb isotope ratio of the uncontaminated soil was far from the Pb isotope ratio of the samples. This suggests that uncontaminated soil has less impact on HMs in barks and leaves. Conversely, anthropogenic sources may be an important contributor to HMs pollution.

**FIGURE 4:**
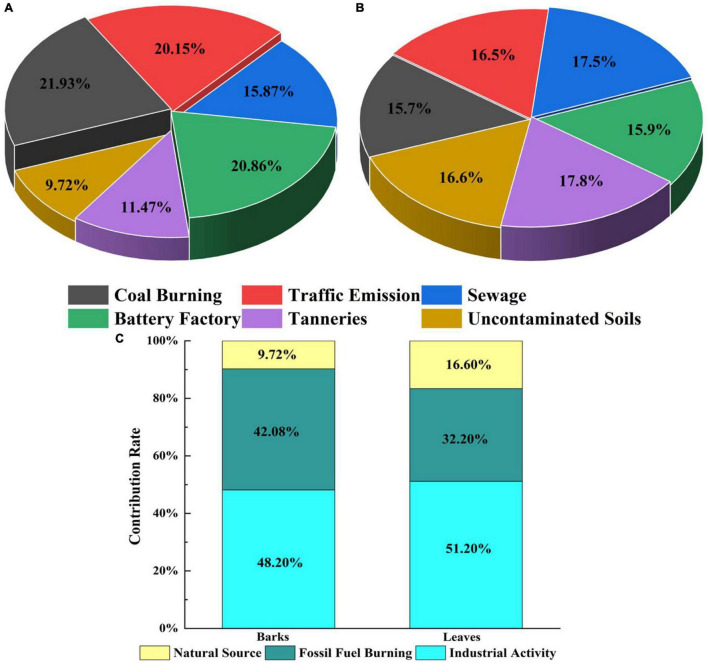
Contribution of different pollution sources to heavy metals (HMs) in barks **(A)** and leaves **(B)**.

The calculation results showed that the influence patterns of different pollution sources on barks and leaves were different (Figure 4). Coal burning, battery factories, and traffic emissions contributed relatively high HMs in barks, all exceeding 20% (Figure 4A). However, the contribution rate of the three to leaves is not a lot different from that of other pollution sources, and they all remain between 15 and 18% (Figure 4B). In order to further improve the efficiency of the initial control of pollution sources, we classified the six pollution sources in the study area into three categories. Classify coal burning and traffic emission as fossil fuel burning; classify sewage, battery factories, and tanneries as industrial activities; and classify uncontaminated soil as a natural source. In general, the order of contribution of pollution sources in barks is industrial activities (48.20%), fossil fuel burning (42.08%), and natural sources (9.72%); the order of contribution of pollution sources in leaves is industrial activities (51.20%), fossil fuel burning (32.20%), and natural sources (16.60%) (Figure 4C).

### Spatial Distribution of Health Risks From Heavy Metal Pollution Sources

**FIGURE 5:**
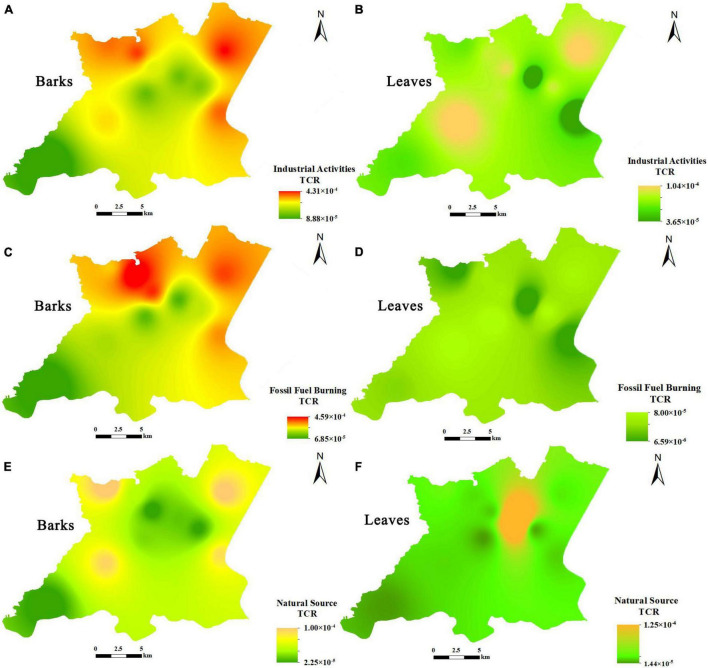
Effects of pollution sources on the carcinogenic risk of camphor barks and leaves.

The carcinogenic risk of camphor tree barks and leaves in the study area caused by different pollution sources is different, but except for Figure 5F (HMs from nature in leaves), the high-risk distribution areas corresponding to each source are basically the same (Figure 5). Among them, the high-risk areas of barks caused by industrial activities are distributed in the northwest, northeast, and southeast of the study area (Figure 5A); the high-risk areas of fossil fuel burning sources are basically the same as the former (Figure 5C); the difference is that it affects the northeast more than the former. This may be due to the fact that there is the G25 “Changchun–Shenzhen” expressway running from the northwest to southeast of the study area. Studies have shown that the combustion of gasoline and diesel can increase the concentrations of Zn, Cu, and Pb in the environment (Fan et al., 2022). Natural sources posed much less risk than the other two sources, with high-risk areas only reaching a threshold for carcinogenic risk (Figure 5E). The high-risk areas for leaves from industrial activities (Figure 5B) and natural sources (Figure 5F) also just exceeded the threshold, and it is worth noting that the high-risk areas for natural sources are distributed in the due north of the study area, which is an urban residential area. In terms of non-carcinogenic risk, the impact of the three heavy metal pollution sources on the entire study area was within an acceptable range, and their relatively high-risk distribution areas were consistent with those with high carcinogenic risk (Supplementary Figure 2).

## Discussion

The concentrations of Zn, Pb, Cd, and Cu in normal plants ranged from 10 to 150, 0.1 to 41.7, 0.2 to 0.8, and 3 to 30 mg/kg, respectively (Padmavathiamma and Li, 2007). The concentrations of Zn, Pb, Cd, and Cu in this study ranged from 24.20 to 250.79, 1.12 to 60.95, 0.04 to 1.07, and 3.87 to 26.33 mg/kg, respectively. Except for Cu, the other three HMs all exceeded the normal range. Some researchers investigated the HMs content in the bark and leaves of camphor trees in Hefei, China. Through comparison, we found that the mean values of Pb and Cd in the bark and leaves of camphor trees in this study were generally consistent with the former (Fang et al., 2021). Typical concentrations of Cd in plants are less than 10 mg/kg (Tomašević et al., 2004) and the concentrations of Cd in this study ranged from 0.04 to 1.07 mg/kg. Overall, some camphor trees in the study area have been contaminated with HMs.

As a tall tree that easily accumulates HMs, the camphor tree can be used to remediate HMs-contaminated soils (Zhou et al., 2019). Therefore, when HMs-contaminated bark or leaves are brought into the market as medicinal raw materials, they may pose a risk to human health. The HMs in the camphor tree enter the human body through human ingestion, which will cause different degrees of non-carcinogenic and carcinogenic risks (Nag and Cummins, 2022). The results of the human health risk assessment showed that the average non-carcinogenic risk of camphor bark and leaves in the study area was within an acceptable range, but its carcinogenic risk was unacceptable, especially for the bark. The average carcinogenic risk of the bark is already close to six times the threshold and some high-risk areas may be higher.

The Pb isotope ratio analysis, as an efficient and accurate method for heavy metal tracer, has been applied to the fields of ecology and environmental science in recent years (Beaumais et al., 2022; Dong et al., 2022). This study innovatively applied Pb isotopes to the source apportionment of HMs in the bark and leaves of the camphor tree and combined the source contribution with the human health risk assessment model to calculate the non-carcinogenic and carcinogenic risks of each pollution source to the camphor tree. The results showed that for bark, industrial activities mainly caused high risk to the northeast and southeast of the study area, while fossil fuel burning mainly caused high risk to the northwest of the study area. High-risk areas from natural sources are distributed around the edge of the study area, but their risk values are much lower than the former. Some studies have found that among different functional areas of the city, the HMs content of the camphor tree bark is the highest in industrial areas and the lowest in commercial areas, which was consistent with the results of this study. However, the average value of HMs in the bark in the commercial area is still much higher than that in this study (Zhang, 2019). The distribution of high risk areas caused by pollution sources to leaves is similar to that of bark, with the difference that the former has a lower risk value. The coastal areas of the West Taihu Lake and the northwestern part of the study area have high health risks, possibly due to the dense distribution of refractories, catalysts, and ceramic manufacturing plants in the area, which produce wastewater and exhaust gases containing large amounts of HMs (Chen et al., 2018a). The G25 Changchun–Shenzhen expressway in the study area is another important factor contributing to the high-risk area mentioned above. Hu et al. (2014) observed that the average concentration of HMs on the main road increased with the increase in traffic flow; Cd and Cu would be generated from automobile engine and brake pad wear, Pb would be generated from automobile exhaust, and Zn would be generated from lubricating oil and tire wear (Yin et al., 2011). In general, the distribution area of camphor bark with high carcinogenic risk in the study area is consistent with the distribution of industry and resources in the area. Therefore, the prevention and control of HMs pollution should be focused on this region.

## Conclusion

This study found that camphor trees in the study area have been contaminated by HMs, and high-risk areas should be avoided when using the barks or leaves of camphor trees in the study area to make Chinese herbal medicine. Pb and Cd are the major HMs that pose increased health risks. Although the THI of barks and leaves was within the acceptable range, the THI of barks was close to 1.0 and the contribution rate of Pb was as high as 56.00%. TCR should be given more attention. The TCR of the bark has been as high as 5.95 × 10^–4^ (>1.0 × 10^–4^), of which the contribution rate of Cd was 48.74%; the TCR of leaves was 1.62 × 10^–4^ (>1.0 × 10^–4^). The HMs pollution in the study area mainly came from industrial activities and fossil fuel burning and the average carcinogenic risk of the two barks reached an unacceptable level: 2.85 × 10^–4^ and 2.54 × 10^–4^, respectively. The areas most affected by various pollution sources in the study area were basically the northwest, northeast, and southeast. This distribution pattern was related to the industry, population, and transportation resources in the study area. The conclusion of this study can provide a reference method for the source apportionment of HMs pollution in higher plants in developed economic areas and also provide a new perspective for environmental management departments to control and prevent HMs pollution of camphor trees.

## Data Availability Statement

The original contributions presented in this study are included in the article/Supplementary Material, further inquiries can be directed to the corresponding author.

## Author Contributions

NL wrote the first draft of the manuscript. All authors contributed to the study conception and design, performed material preparation, data collection, and analysis, commented on previous versions of the manuscript, read, and approved the final version of the manuscript.

## Conflict of Interest

The authors declare that the research was conducted in the absence of any commercial or financial relationships that could be construed as a potential conflict of interest.

## Publisher’s Note

All claims expressed in this article are solely those of the authors and do not necessarily represent those of their affiliated organizations, or those of the publisher, the editors and the reviewers. Any product that may be evaluated in this article, or claim that may be made by its manufacturer, is not guaranteed or endorsed by the publisher.
