# Supplementary material for: Source Apportionment and Health Risk Assessment of Heavy Metals in Endemic Tree Species in Southern China: A Case Study of Cinnamomum camphora (L.) Presl
Source: Front Plant Sci. 2022 Jul 11;13:911447. doi: 10.3389/fpls.2022.911447 (PMC9313620; doi:10.3389/fpls.2022.911447)
Supplement: Supplementary file 1 [file Data_Sheet_1.docx]

**Source apportionment and health risk assessment of heavy metals in endemic tree species in Southern China: A case study of *Cinnamomum camphora* (L.) Presl**

Ning Li ^1^, Yan Li ^1,2,^*, Shenglu Zhou ^3^, Huanchao Zhang ^1^, Genmei Wang ^1^

^1^ College of Forestry, Nanjing Forestry University, Nanjing, Jiangsu, China;

^2^ Key Laboratory of Geographic Information Science of the Ministry of Education,

School of Geographic Sciences, East China Normal University, Shanghai, China.

^3^ School of Geography and Ocean Science, Nanjing University, 163 Xianlin Road, Nanjing, Jiangsu, China

* Corresponding author.

E-mail: lyle@njfu.edu.cn

Tel: +86-15005160852

Fax: +86-25-85428629


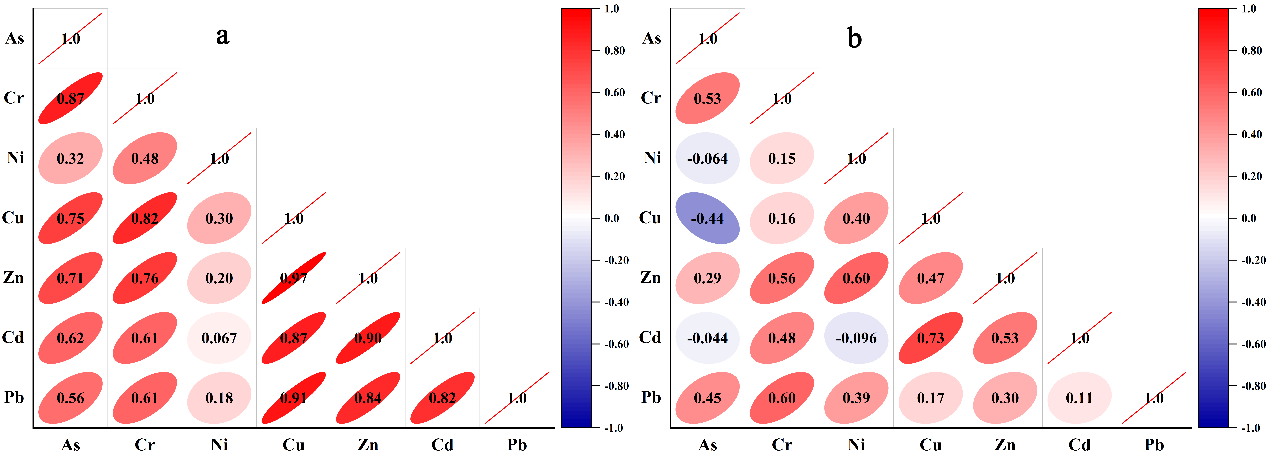


Fig. S1. Correlation analysis of HMs in barks (a) and leaves (b)


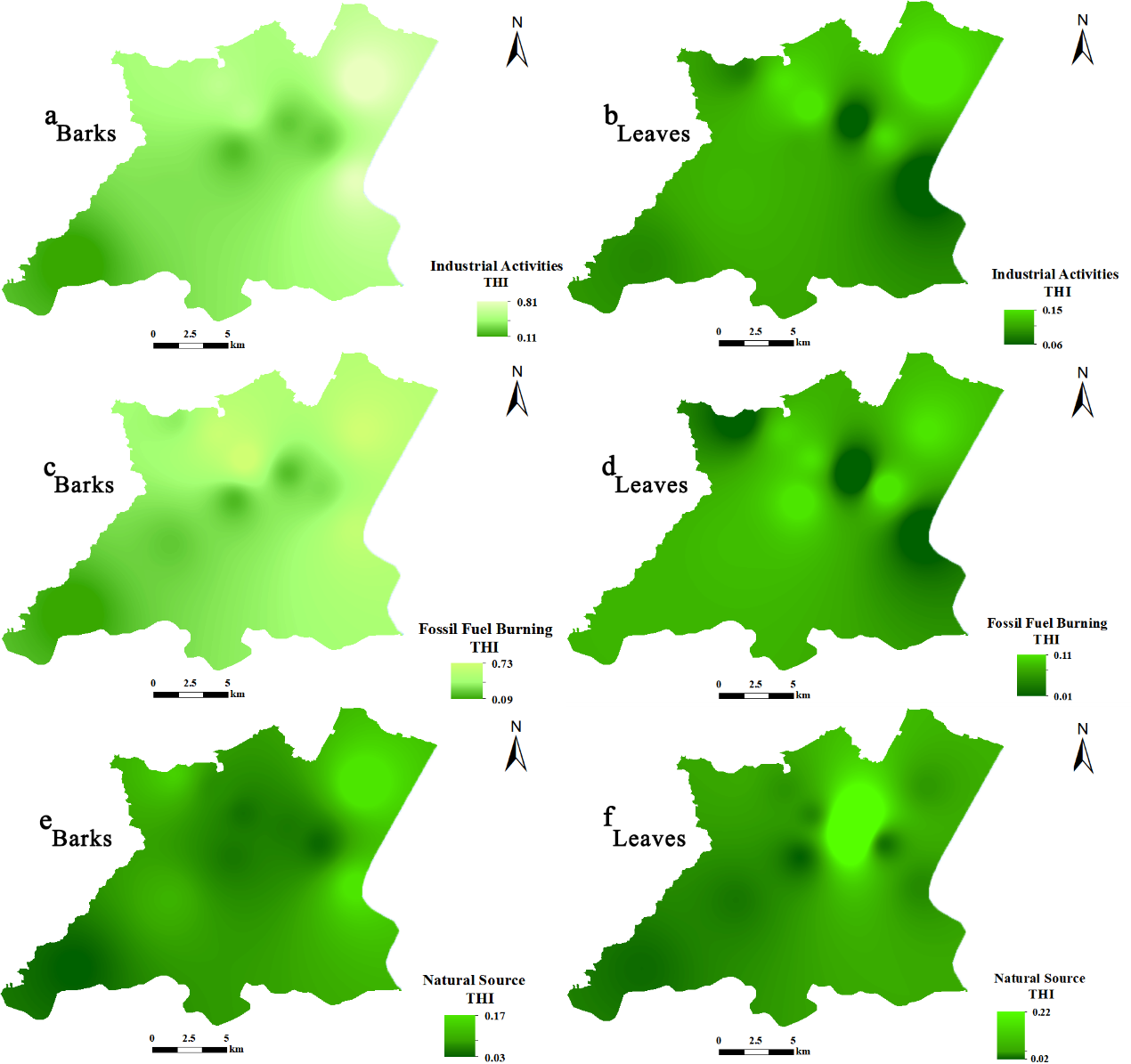


Fig. S2. Effects of pollution sources on non-carcinogenic risk of camphor barks and leaves in the study area

Table S1 The values of parameters for the calculation of health risk

| Parameters | Value | Unit |
| --- | --- | --- |
| IR | 0.015 | kg/d |
| EF | 90 | d/a |
| ED | 20 | a |
| BW | 60 | kg |
| AT | ED*365 | d |

Table S2 RfD and SF values of different heavy metals

| Metals | RfD (mg/kg/d) | SF (kg·d/mg) |
| --- | --- | --- |
| Cd | 0.0005 | 6.3 |
| Cr | 1.5 | 0.5 |
| Cu | 0.04 | - |
| Ni | 0.02 | - |
| Pb | 0.0035 | 0.0085 |
| Zn | 0.3 | - |
| As | 0.0003 | 1.5 |

Table S3 Pb isotopic composition of known anthropogenic and natural sources

| Category | Source | ^208^Pb/^206^Pb | ^206^Pb/^207^Pb |  |
| --- | --- | --- | --- | --- |
| Anthropogenic sources | Traffic emission | 2.1075 | 1.1655 | (Gao et al., 2004) |
|  | Coal combustion | 2.111 | 1.163 | ([Tan et al., 2006](#_ENREF_1)) |
|  | Sewage | 2.098 | 1.1715 | (Hu et al., 2013) |
|  | Battery factory | 2.109 | 1.158 | (Hu et al., 2013) |
|  | Tanneries | 2.084 | 1.18 | (Hu et al., 2013) |
| Natural sources | Uncontaminated soils in eastern China | 2.079 | 1.197 | ([Zhu et al., 2001](#_ENREF_2)) |

**Reference**

Gao, Z.Y., Yin, G., Ni, S.J., 2004. Geochemical feature of the urban environmental lead isotope in Chengdu city. Carsologica Sinica. (In Chinese)

Hu, G., Yu, R., and Zheng, Z., 2013. Application of stable lead isotopes in tracing heavy-metal pollution sources in the sediments. *Acta Scientiae* Circumstantiae. 33, 1326-1331. (In Chinese)

Tan, M.G., Zhang, G.L., Li, X.L., Zhang, Y.X., Yue, W.S., Chen, J.M., Wang, Y.S., Li, A.G., Li, Y., Zhang, Y.M., Shan, Z.C., 2006. Comprehensive study of lead pollution in Shanghai by multiple techniques. Anal. Chem. 78, 8044-8050. <http://doi.org/10.1021/ac061365q>.

Zhu, B.Q., Chen, Y.W., Peng, J.H., 2001. Lead isotope geochemistry of the urban environment in the Pearl River Delta. Appl. Geochem. 16, 409-417. <http://doi.org/>10.1016/S0883-2927(00)00047-0
